# Examining the Research Evolution on the Socio-Economic and Environmental Dimensions on University Social Responsibility

**DOI:** 10.3390/ijerph17134729

**Published:** 2020-07-01

**Authors:** Víctor Meseguer-Sánchez, Emilio Abad-Segura, Luis Jesús Belmonte-Ureña, Valentín Molina-Moreno

**Affiliations:** 1 International Chair of Social Responsibility, Catholic University of Murcia, 30107 Murcia, Spain; jvmeseguer@ucam.edu; 2 Department of Economics and Business, University of Almeria, 04120 Almeria, Spain; lbelmont@ual.es; 3 Department of Management, University of Granada, 18071 Granada, Spain; vmolina2@ugr.es

**Keywords:** university social responsibility, economic, environment, corporate sustainability, sustainable development, scientific production

## Abstract

Responsible higher education institutions have an impact on society and economic, environmental, and social development. These effects define the axes of the socially responsible management of the universities. The concept of university social responsibility (USR) manages these relationships to produce a positive impact on society through higher education, research, and the transfer of knowledge and technology, as well as education for sustainability. For this study, worldwide research into this subject was studied for the period 1970–2019. A bibliometric analysis of 870 articles was made, obtaining results for the scientific productivity of the journals, authors, institutions, and countries contributing to this research. The main category is business, management, and accounting. The most productive journal is the Business and Society Review, while the California Management Review is the most cited. The authors with the most articles are Stavnezer, Luo, and Lanero. The most productive institution is Wuhan University. The United States is the country with the most publications and citations, and the same country, together with the United Kingdom, make the most international contributions. Evidence shows growing worldwide interest in the economic and environmental impacts of USR. Future research should focus on analysing the links between the responsible and sustainable consumption of universities and their short-term financial, economic, and sustainable impacts.

## 1. Introduction

In recent decades, the impact of global changes, both economic and political, social, environmental, and technological, has led to a shift in the role of higher education institutions (HEIs) in contemporary society [1,2]. In this context, in the process of value creation, organizations, and amongst them universities, are obliged to manage intangible assets and corporate values. Thereby, in the education sector, institutions need to link ethics and responsibility to quality and sustainability in their mission [3].

Currently, the survival of universities is related to the social and environmental implications of their actions. In this sense, the transfer of knowledge, associated with economic growth and social development, has a place in public and economic policy [1,2,3].

Thereby, the social responsibility of universities includes education, organization, cognition, equity, and socialization, to create more sustainable societies [4]. Amongst its contributions in this respect are the fight against social inequality and corruption, employment, economic development, dynamism, and community initiatives, as well as the implementation of technological advances [5].

Since the classical definition of the university as a community of learning and teaching, knowledge has become one of the main instruments for the development of societies [6,7]. For this reason, society requires more knowledge creating universities as learning communities [8]. Consequently, HEIs have contributed to societies or economies of knowledge from different perspectives, such as politics, economics, or research, among others.

To the initial missions of universities, which were teaching and research, a third has been added related to community commitment. This “third mission” has drawn attention to university institutions on the part of international agencies and political leaders [9], promoting policies with a social impact on societies. This has meant that, to a certain extent, the central axis of this mission will guide the perspective of the main activities of universities, changing the functioning and management of traditional academia.

The concept of university social responsibility (USR) is based on the statements of the stakeholder’s theory, in order to unify the commitment that it acquires to fulfil the expectations of the different groups in their economic, social, and environmental functions.

In this context, the revised literature points to terminological inaccuracy concerning the main concept of research. USR is a concept that derives and connects with the terms Social responsibility, corporate social responsibility (CSR), sustainable development, corporate environmental responsibility (CER) and education for sustainability, and not with terms related to philanthropy [10,11].

Thereby, with some generality, USR is a commitment of the university institution to develop initiatives that promote its relationship with the different social groups of society. Similarly, it is considered university management policy to promote actions that improve this relationship between the university and society, to balance the impact at both academic and organizational level [12]. Therefore, globally, universities haves been making great efforts in recent years to prevent the advances made in ethical terms and social transformation from being confused with a series of good practices of little transcendence and, instead, to be considered as a policy of transversal management of their activities [13].

As regards putting USR into practice, it can be considered within the regulatory framework of quality management in relation to the commitment of universities to meet the demands of knowledge transfer and research results [14]. In particular, the international standard ISO 9001 certifies the quality management system of universities in terms of the organization and planning of activities, establishing, among others, the criteria for controlling and monitoring the processes involved [15]. For its part, ISO 26,000 mentions social responsibility in HEIs from a managerial perspective: the focus of university activities themselves towards achieving a sustainable and healthy society; and at a normative level, in terms of guiding responses to the demands posed by different interest groups in terms of mutual benefit [16].

A quality education is the basis for improving people′s lives, as well as for sustainable development. Great progress has been made in access to education at all levels and, similarly, in enrolment rates at universities, particularly for girls and women. Although basic literacy has increased markedly, greater efforts are needed to achieve the goals of universal education and achieve equality for women in the university and their insertion in technical careers.

In this sense, the quality management of HE is linked to sustainability and the development of a peaceful and equal society. These aspects, in turn, influence the generation of a higher quality of life and a greater positive impact on the environment, on the welfare state and on the economy in general [7,10].

Likewise, in the last decades, universities have gone from being dependent on centrals governments, elitist, small, closed and with little scientific production, to reverse this situation and be autonomous, decentralized, internationalized and more active in research, in general lines. This evolution has also meant that the institution participates in the environment in which it is located and cares about the social, economic, and environmental aspects in a productive way, generating innovation and added value [8,12,14].

The review of the literature, on the other hand, has found different studies that discuss whether universities, as institutions committed to sustainable development, use the USR as an instrument to favourably manage their reputation in their financial statements [17] or, on the contrary, play a prominent role in social responsibility, providing information with sufficient transparency [18,19].

In addition, there is the controversy of equating the CSR with the USR, since the former was formed as a concept of market requirements for business organization, while from a university perspective, it emerged in response to the social needs linking its development with knowledge transfer from HEIs [20,21].

This has justified the interest to develop this research. Hence, the motivation of this study is to document the evolution of the knowledge base of the economic and environmental impacts of USR, with the intention of contributing to educational institutions to the approach of sustainable consumption, in relation to minimizing the environmental impact and satisfying consumer needs, both for present and future generations.

From the reviewed literature, the research questions have been identified, which refer to determining what is the structure of knowledge about the economic and environmental impacts of USR; which are the most productive authors, institutions and countries; and what are the thematic axes that this research topic develops and towards which they are evolving.

One of the main limitations of this study is to discern whether, among other variables, the number of publications related to a particular community is due to the demands of given interest groups, or on the contrary, to the needs of society.

Accordingly, the main objective of this study is to analyse research trends on the economic and environmental impacts of USR globally over the period 1970–2019, i.e., over the last fifty years. This period ranges from the first article published on this topic (1970) to the last full year (2019). The study is consequent in admitting that the extensive period analysed covers numerous global socials, economic and political changes, which have involved different approaches in the management of university campuses. Furthermore, the appearance of new concepts has modified the analysis in scientific research on this topic.

To answers these research questions, a sample of 870 selected scientific journal articles from the Elsevier Scopus database was analysed. The bibliometric method was followed to synthesize the knowledge base on the implications of USR in a global context.

Finally, it should be noted that the research lines currently being developed on the subject include, among other things, the relation between the effect of implementing social responsibility policies in public and private universities in different countries with different political and economic systems. In addition, different studies are being related that analyse the outcome of social responsibility of HEIs with policies of inclusion and diversity.

## 2. Scope of Research

The study of USR is based on the theoretical principles of interest groups analysis, which, together with the basic concepts, define the reference framework in this field of research. Hence, these explanatory principles define how a set of phenomena behaves, to systematise and generalize individual cases. Consequently, with the study carried out, Section 2 is the result of the analysis of the reviewed literature and supposes a framework and a guide on the research topic, on which the following sections are based.

### 2.1. Backgrounds

In 1930, Ortega y Gasset, in his work “Mission of the University”, and E. Allen Gaw, in his publication “Social Education”, discussed of the roles that university institutions needed to play in order to participate in the process of modernising society, and proposed, in a debate still valid today, that higher education (HE) should be reformed for the benefit of society [22,23,24].

In 2003, at the World Conference on Higher Education, UNESCO stressed the importance of the well-being of nations and the quality and scope of HEIs. Since then, arguments and definitions have flowed that make it possible to distinguish the practices and policies of social responsibility related with universities from those that mainly relate to companies.

In particular, among the purposes pursued by HEIs, are, in politics, the development of democracy and conflict resolution [25]; in the economy, sustainable economic development [26]; in social terms, the well-being of people and their integration, solidarity and the culture of the common good; in education and research, capacity-building, and knowledge advancement [27]; and finally, in ecology, health, equity, wellbeing, and environmental sustainability [28].

Universities today have a complex end goal, whereby they must respond to confusing questions of strategy. These strategies and alternatives should be aimed, amongst other things, at analysing the causes of the expansion of HE [29], to seek the balance between equality and excellence [30], and to reconcile the close relationship between the university itself, the government and the market, since the university′s ultimate mission is to internalize that it is the area where strategies are developed to strengthen future benefit options, as well as where market skills and competencies are acquired [31].

In this sense, the triple helix model is a valid framework for describing interactions and relationships between universities, industries, and governments, with a view to capitalizing on knowledge [32,33]. Therefore, in addition to traditional education and research missions, according to this model, universities enable the usefulness of knowledge by organizing technology transfer and business activities [34].

Table 1 presents the main results on the state of the subject of research, ordered chronologically, i.e., the economic–environmental impacts of the USR in a global context, after the analysis of the reviewed literature.

The literature review has made it possible to analyse documents that are what have allowed us to define the bases of this research. Table 1 displays only the main articles of the reviewed literature, which, as mentioned, initially allowed the formulation of the research question and the objective of the study, in addition to selecting the search terms in the applied methodology.

### 2.2. Theoretical Framework

This subsection examined the theoretical principles used to conceptually argue the link between social responsibility, sustainable consumption, corporate sustainability, and HEIs [50,51,52]. Therefore, the purpose of this section is to justify the theoretical principles that support the economic and environmental impacts of USR, for its conceptual definition.

The concept of USR must be established according to the axioms of stakeholder theory to unify its commitment to meeting the expectations of the different stakeholders in their health, economic, social, and environmental functions [53,54].

First, the theoretical approach of stakeholders, enunciated in 1984 by Freeman [55], is established, although, previous to this, a need was expressed to explain the active contributions of corporate social responsibility [56]. Interest in CSR has increased with growing demands for companies to take responsibility for their social impact and to serve the general interest [57], and not just the minority of stockholders [58]. While these changes were initially intended to include environmental and social objectives, at least companies have taken an interest in how they interact with stakeholders [59].

On the other hand, recent decades have seen a growth in the influence of stakeholders on organizations, but to successfully implement and manage social responsibility, there is a need to know and identify their needs and expectations. The implementation of stakeholder theory in HEIs in order to create knowledge-based societies led to the need to promote university policies in social responsibility based on participatory methods, and thus contribute to the liaison between academic and research functions, and social needs and problems [60,61].

For all these reasons, in recent decades, universities have gone from being dependent on central governments, elitist, small, closed and with little scientific production, to reverse this situation and be autonomous, decentralized, internationalized and more active in research, in lines general. This evolution has also meant that the institution participates in the environment in which it is located and cares about the social, economic, and environmental aspects in a productive way, generating innovation and added value [54,60].

### 2.3. Conceptual Framework

**Figure 1:**
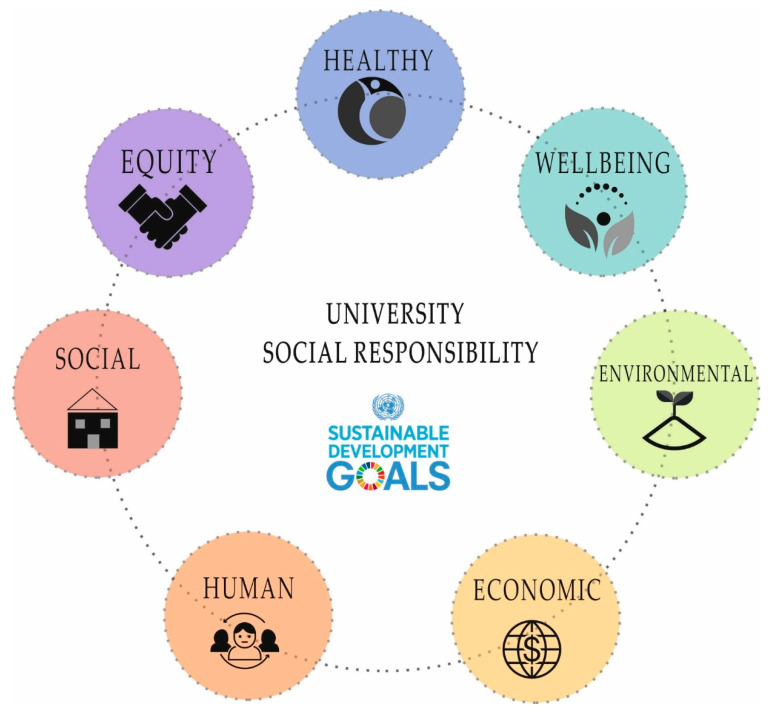
Conceptual structure of USR.

The revised literature provides definitions for the basics of this research topic. Some reflections are included on the terms and concepts used in the context of this research, which have helped model the term university social responsibility (USR) from the idea of corporate social responsibility (CSR). Hence, Figure 1 shows the conceptual structure of the study topic, that is, it includes the terms of the CSR and USR, which respond to the literature review and the specific SDGs in the research.

Likewise, although the official definitions of CSR refer mainly to companies, the general consensus is that the foundations of CSR affect all types of organizations, be they companies, public administrations, labour unions, or NGOs among others. Furthermore, it is a voluntary initiative of management and good governance, which, integrated from the decision-making of an organization, takes into account its stakeholders, and economic, social, and environmental variables. For these reasons, the USR is the answer that the university must train responsible citizens with their environment, creators of creative ideas and committed to help solve social, economic, and environmental issues.

Since its initial definition, the concept of the CSR has not undergone major changes, although its role in company strategy has evolved, as has the effect that socially responsible actions had on sustainable development. The term emerged as a response of companies to growing social and corporative concerns with issues that included social, environmental and consumer interests.

Since its inception, CSR has been extensively studied and researched [62,63,64,65] from several angles, such as its impact on financial outcomes, the competitiveness of the company or organization or its relationship with sustainability [66,67,68], to offer a comprehensive concept. Today, the principles of CSR engagement continue to generate debate in the business-related literature, and, in the same way, these issues influence the activities of the university as an organization. This is broadly due to the fact that universities, as a paradigm of the knowledge society, are being more closely observed in terms of their social responsibility. This is particularly true with the fall in public funding, such that universities are increasingly run as commercial and competitive institutions faced with wider access to education [5,42].

As regards social responsibility, institutions should treat laws and regulations as opportunities for improvement, insisting on ethical behaviour in their interactions with stakeholders [69]. In this way, the social impact of universities institutions must arise from ethical practices by defining a number of indicators to ensure that responsibility meets the basic requirements.

For its part, the concept of sustainability formally appeared for the first time in the Brundtland Report in 1987 [70], related to the environment and development. The term refers to meeting today′s needs without compromising options in the future [71]. Later, in 1998, J. Elkington [72] introduced the concept of sustainability as the triple bottom, including financial, social, and environmental aspects in the results of a company or organization [73,74,75]. In this way, the economic value, the degree of social responsibility and the environmental impact of a company are brought together.

Furthermore, just as companies and other organisations have recognised the demand for greater social and environmental sustainability, universities have responded with new pedagogical approaches. Hence, arose the concept of education for sustainable development (EDS), a term that refers to the various forms of learning and teaching associated with the sustainable development of societies [76]. In this way, UNESCO describes how EDS trains students to make responsible decisions in terms of environmental integrity, economic viability, and a just society, for present and future generations, leading the positive transformation of society [77].

In relation to the basis of sustainable development and the Sustainable Development Goals (SDGs) by 2030, education allows for self-reliance, fosters economic growth by improving skills and improving people′s lives. Thus, the USR and stakeholders require better access and quality to the university, eliminating, among others, gender inequalities, food insecurity and armed conflicts [78].

In addition, by bringing together these concepts that address social responsibility from different perspectives, it is noted that, from their origin, universities have been the basis of education and, to some extent, of social sustainability. In this context, universities are obliged to implement a series of ethical principles and values in the fields of management, teaching, and research, so that they declare their intentions, in this sense, in their vision and mission [20,79,80].

Hence, in the knowledge society, USR refers to managing the strategic model in the context of a socially responsible HEI. For this reason, the implementation of the USR entails self-criticism from the institution itself, responsibly assessing the impact of its actions on society [81,82]. That is, the transformative function of the institution requires that it modifies its structure in order to articulate its mission of providing a service to society [83,84].

As mentioned by A. Parsons [85] of the UNESCO Chair for Community Based Research and Social Responsibility in Higher Education, it is necessary to recognize that the USR concept focuses on the democracy of the university′s internal processes, respect of the university for the environment and whether the institution designs programs to produce socially responsible graduates and by implementing programs that involve community engagement.

This social concept reviews the actions of universities in a knowledge society to determine the applicability of CSR in these educational institutions. At the same time, USR encompasses the operational variables that a university must manage if it is to be socially responsible in terms of support for inclusion, community education, and respect for and conservation of the environment or natural resources [10,13].

### 2.4. Regulatory Framework

As regards the USR regulatory framework, the international standard ISO 9001, which deals with the quality of management systems and accreditation, certifies universities in terms of optimal processes of organization and planning of activities. ISO 26,000 establishes the management and focus of social responsibility in universities from a managerial perspective to achieve a sustainable society [15,86].

The Bologna Process (1999), the basis of the subsequent European Higher Education Area (EHEA), which is organized on the principles of quality, mobility, diversity and competitiveness, was initially directed towards increasing employment in the European Union and the transformation of the European higher education system into an instrument for attracting students and teachers on a global scale. Thereby, the incorporation of the social dimension of HEIs was not initially considered. Subsequently, the following statements have examined the influence that universities have on the development of European society [25,87,88]. In this respect, the Council of Europe emphasized the public responsibility of HE in relation to development of its social dimension.

Therefore, in 2015, with the support of the European Union′s Lifelong Learning Programme, the Comparative Research Project on University Social Responsibility in Europe and the Development of a Community Reference Framework (EU-USR) were initiated. On the initiative of certain leading European Union universities regarded as leaders in social responsibility, the objective of this common European model was to optimise the USR of all universities. Initially, it was formed in response to the European Commission′s political priority on the need for a common social responsibility strategy for all European universities. Today, many European universities understand their social dimension, promoting policies and practices, albeit without a frame of reference [89].

Likewise, both the Millennium Development Goals, in 2000, and the SDGs, in 2015, mainly in SDG 4, have promoted, in relation to the USR, an inclusive, equitable and quality education that promotes learning opportunities during all life for all. In this way, these resolutions establish the bases for the achievement of a quality education as a foundation to improve people′s lives and sustainable development.

Table 2 presents the various international bodies that have provided definitions, policies, regulations, normative and practices to integrate the concept of USR, ordered chronologically.

In this sense, the European Commission managed the INHERIT project (INter-sectoral Health and Environment Research for InnovaTion) to stimulate effective policies, practices and innovations in relation to key environmental stressors and the underlying causes of inequity in health. Likewise, the objective of this Horizon 2020 research project, for the period 2016–2019, was to promote the change of sedentary lifestyles and those outside the circular economy, and thus formulate scenarios to create a more sustainable future. In other words, this project sought to design and implement intersectoral initiatives to achieve healthier social change [90].

## 3. Materials and Methods

### 3.1. Bibliometric Method

Scientometrics is the science that studies and measures scientific production. It is also known as the scientific and empirical study of science and its results. In practice, there is a significant overlap between scientometrics and other scientific fields: bibliometric, information system, information science and scientific policy [91]. It is based mainly on the works of Dereck J. de Solla Price and Eugene Garfield, who founded the Institute for Scientific Information (ISI) in 1960. Later, in 1998, Garfield created the journal Scientometrics.

Bibliometric, on the other hand, applies mathematical and statistical methods to the literature of a scientific nature and the authors that produce it, with the aim of studying and analysing scientific activity. The instruments used to measure aspects of scientific activity are bibliometric indicators, which are measures that provide information on the results of scientific activity in any of its manifestations [92]. It was introduced by E. Garfield in the mid-20th century, and has since become widespread in scientific research and, for decades, has helped to review knowledge across multiple disciplines. Hence, scientometrics, together with bibliometric, has evolved from reflections on scientific development and the availability of numerous databases for researchers.

### 3.2. Data Collection and Processing

The objective of this study is to provide an insight into the overall dynamics of research and the state of the issue regarding the economic-environmental impacts of the USR in a global context. To achieve the proposed objective, a quantitative analysis was carried out, using bibliometric, to identify, organize and analyse trends in the research topic. In recent decades, it has contributed to the review of scientific knowledge, and has been successfully used in various scientific fields [93,94].

According to the reviewed literature of the study topic, mainly the one detailed in Table 1, the terms chosen in the search string were “corporate social responsibility”, “CSR”, and “university”.

The choice of the Scopus database is due to the fact that, when performing the initial search in the Web of Science (WoS) and Scopus databases, it showed a significant difference in the volume of articles in the analyzed period 1970–2019, since 559 articles were extracted from WoS, and 870 articles from Scopus.

Additionally, Scopus has greater advantages over other databases, such as WoS or Google Scholar, among which are: (i) it is considered the largest repository of peer-reviewed literature; (ii) it minimizes the risk of losing documents during the search; (iii) it is easily accessible and offers some tools for data visualization and analysis, in addition to presenting the option of downloading content in different formats; and, (iv) it presents a variety of data.

**Figure 2:**
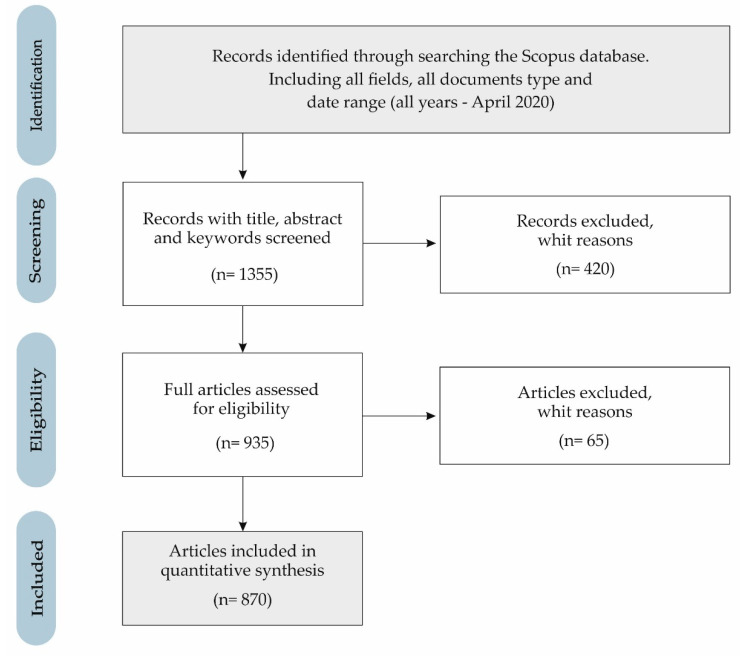
Flow diagram of the sample selection (PRISMA).

The process followed in the selection of the sample of documents conforms to the flowchart of Figure 2, according to the preferred reporting elements for systematic reviews and meta-analyses (PRISMA) [95].

Thereby, in phase 1 (identification), 249,478 records were identified from the Scopus database, considering all fields for each of the key search terms (corporate social responsibility, CSR, and university), all types of documents, and all years in the data range (all years - April 2020).

In phase 2 (screening), the option of “article title, abstract and keywords” was chosen in the field of each term, so that 248,123 records were excluded, so 1355 records were identified.

Subsequently, in phase 3 (eligibility), only the articles were selected as the type of document, to guarantee the quality derived from the peer review process. In this phase, 420 documents were excluded, so it had 935 records.

As for the time horizon of analysis, this is between 1970 and 2019, both included, that is, from the publication of the first article on this topic (1970), to the last full year (2019). For this reason, in the last phase (included), 65 documents are excluded, so the final sample included 870 articles.

In short, the search selected records of subfields of title, abstract and keywords, in the period that contains the last 50 years, from 1970 to 2019, in the same way that it has been successfully applied in other studies that have used the bibliometric methodology [96,97,98]. The representation of this sample of documents is supported by the proven quality of the Scopus database, with respect to the indexing protocol, in addition to the systematic procedures of the search criteria.

Therefore, the final sample included a total of 870 articles, both open access and non-open access. The variables analysed were year of publication, thematic area, journal, author, author’s country of affiliation, institution to which the author is affiliated, and keywords that define the publication.

In this study, the indicators of scientific production analysed were the distribution by years of published articles, the productivity of authors, countries, and institutions [99]. As for the quality indicators that refer to the impact of the different authors in the research field, we used: the h-index, the number of citations, and the indicator that measures the quality of the scientific journals included in Scopus, SCImago Journal Rank (SJR).

In addition, collaborative structure indicators, which measure the links between authors and countries, were analysed through network mapping and processing tools due to their reliability and suitability in the bibliometric analysis.

VOSviewer software (version 1.6.15., Leiden University, Leiden, The Netherlands) was applied for the analysis of collaboration structure and relationship indicators, by co-authorship and co-occurrence analysis, which provides data on interactions and the evaluation of subject-matters, to measure the activities of research networks. In other words, this tool allows analysis for the purpose of displaying relationship maps and network links between authors, institutions, countries, and keywords. Subsequently, this software tool allows you to recognize research trends based on the use of keywords in research articles. In this sense, network mapping refers to the process of seeing the connection and structure of the network in order to map the dynamics of science, in this case, of a certain research field, and which seeks to explore the structure underlying similarities and interrelationships between documents [100,101].

Likewise, the clustering algorithm of VOSviewer allows communities to be detected in a network that considers modularity, that is, a measure that assesses the quality of community structures. Thereby, modularity-based clustering of VOSviewer provides networks where nodes are densely connected internally within groups, but without external connection between different groups. In this way, it unifies the mapping and grouping approaches, in addition to dividing the research carried out into documents [102]. The results obtained are useful for researchers, academics, analysts, managers, and other stakeholders since scientific activity in this research field is evaluated.

## 4. Results and Discussion

This section presents and discusses the results of scientific production concerning the economic-environmental impacts of USR in a global context, as well as the distribution of publications by subject area and journal, and the scientific production of authors, institutions, and countries. The results obtained from the analysis of the keywords associated with this field of research are also discussed.

### 4.1. Evolution of Scientific Production

Table 3 presents the evolution, from 1970 to 2019, of the main features of the articles published on economic-environmental impacts of the USR. During this period, it is noted that interest in USR research increased, especially in the last decade, as seen from the variables analysed. For example, in the period 1970–1979 only seven articles on this subject were published, while in the last decade analysed (2010–2019) the number reached 743, that is, 106 times more. The increase in the amount of publications in the last decade is emphasised by the fact that 85.40% of the total number of analysed articles were published in this period. The year in which the most contributions (118 articles) were published was 2019.

**Figure 3:**
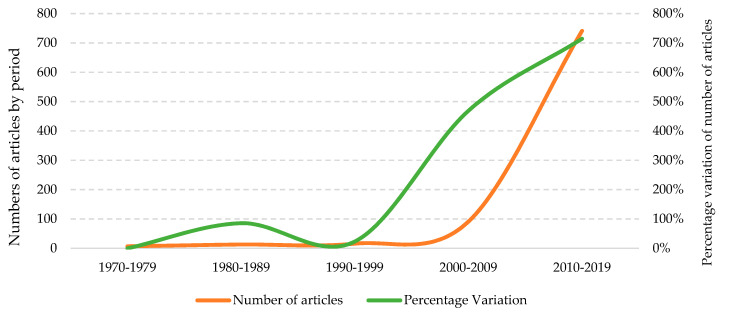
Evolution of the number of articles and percentage of variation (1970–2019).

Figure 3 shows the evolution in the number of articles and their percentage of variation between each decade studied. In addition to the considerable increase in the number of articles published over the past 10 years, the percentage growth produced in the fourth (468.80%) and fifth (714.30%) period analysed, that is, in 2000–2009 and 2010–2019. The percentage increase in the number of publications in 2010–2019 is due to the fact that this was the first decade in which more than 100 articles (741) were exceeded, while in the previous decade (2000–2009) only 91 articles had been published.

As with the number of articles, the total number of authors also increased during the period analysed. In the last decade (2010–2019), 85.70% of the total number of authors for the whole 50-year period published at least one article. While only 11 authors published in this field of research in the first decade (1970–1979) a total of 2001 authors were active in the period 2010–2019. The average number of authors in the third and fourth decades was 2.8 authors per article, while in the last period studied it was 2.7 authors per article.

The number of countries involved in the publication of articles on this research subject increased from 2 in the first decade, 1970–1979, to 86 in the last period analysed, representing 95.56%. During the whole period analysed, 90 countries contributed to the publication of articles on the scope of USR.

In addition, the number of citations grew exponentially, from the first period (2001–2003), increasing from 813 to 5737 in the last period analysed (2010–2019). It should be noted that the articles corresponding to the most recent decades will receive a greater number of citations in coming years, both because of their recent publication and impact, and for their distribution in open-access [103]. This is related to the average annual number of citations per article, which decreased from 116.1 in the first period (1970–1979) to 7.7 in the last period (2010–2019).

Finally, the number of journals where USR articles were published increased from 2 in the first period, 1970–1979, to 397, in the last decade analysed, 2010–2019, which also accounts for 88.03% of the total number of journals examined in the 50-year period.

Scientific production on the research topic, influenced by the link with the concepts of corporate social responsibility and the HEI at a global level, was developed in line with the technological advance that brought about the appearance of the Internet globally, leading to greater ease in the use of data and international academic and research cooperation [30,91,98].

In addition, articles published in the English language account for 92% of the total, as the main language of science. Here, English is followed by Chinese (4%) and Spanish (2%). The remaining languages do not add up to 1%.

### 4.2. Distribution of Publications by Subject Area and Journal

During the period analysed, 1970–2019, there are several categories in which works related to the economic and environmental impacts of USR can be found. In this way, according to the Scopus classification, there are a total of 26 thematic areas into which the 870 articles analysed are classified. It must be borne in mind here that the same article can be classified in more than one category, depending on the interest of the author and the publisher.

**Figure 4:**
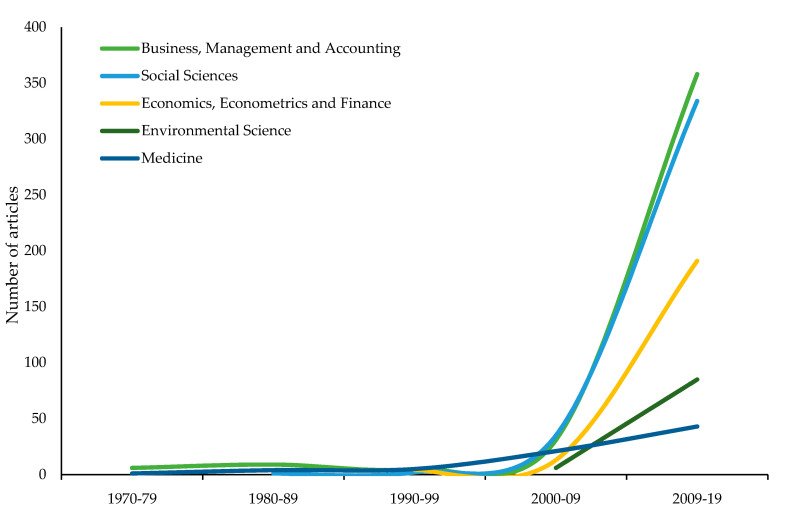
Comparisons of the growth trends of subject areas (1970–2019).

Figure 4 shows how the thematic classification of articles on the subject of study has evolved in recent years. Business, management, and accounting predominate throughout the period studied, with 27% of all published articles on USR falling into this category. These are followed by, in order of importance, the social sciences (25%), economics, econometrics, and finance (14%), environmental science (6%), and medicine (5%). Thus, the five most important categories represented in Figure 2 cover 76% of the documents published in this field of research from 1970 to 2019. Except for arts and humanities (4.2%) and engineering (4%), the remaining subject areas do not reach 2% of all published works.

The association of publications in this field of research, mainly the categories of Business, Management and Accounting, and Social Sciences, arises from the conceptual and practical link of the dialogue between the USR and the stakeholders, about the economic, social, and environmental factors [46,51].

Moreover, as can be seen in Table 2, there has been an increase in regulations since 2000, which has been reflected in the increase in the volume of articles that are associated with the Business, Management and Accounting and Social Sciences subject areas. Thereby, it is observed that the evolution over time of the regulatory framework has affected certain thematic areas of knowledge.

The important changes that have taken place in these five decades, both socially, economically, politically, culturally and environmentally, have obviously motivated scientific production to evolve, since the researcher and academic seek and need to find answers to these changes. Along these lines, the Millennium Development Goals, in 2000, and the SDGs, in 2015, mainly in SDG 4, stand out mainly in the promotion of quality education [38,51,70].

Table 4 shows the main features of the articles in the 20 journals with the highest number of published articles on economic and environmental impacts of USR in a global context. Of note is the high percentage (45%) of journals belonging to the first quartile (Q1) of the 2018 SJR index, SCImago Journal Rank. As can be seen, USR studies have gradually interested more journals and more academics, as evidenced by the growth in the number of articles and the variety of journals concerned.

By country, among the 20 most important journals in the USR publication are those of European origin: United Kingdom (7), Netherlands (3), Lithuania (2), Switzerland (1), Poland (1), Czech Republic (1), Romania (1), and those of American origin (4).

The journal that has published most articles on the economic and environmental impacts of USR globally is Business and Society Review, with 50 articles, which represents 5.75% of the total number of articles published from 1970 to 2019.

The California Management Review is the journal that has occupied the first position in the ranking for more decades, two of the five periods analysed (1970–1979, 1980–1989). It also stands out because it attracts great interest from the scientific community, as seen by the high number of citations that it received (2098), and for the average number of citations per article (87.42). It is also the journal that has the highest H index for published articles on USR, with 15, which is far from the journal’s overall H index for all subjects, which stands at 118. The journal Nucleic Acids Research has the highest impact factor SJR, 8.636 (Q1), followed by the Journal of Experimental Medicine, with 7.941 (Q1), and the California Management Review, with 2.658 (Q1).

It is important to mention how the journal Sustainability, with 13 articles published on the subject since 2015, has become the journal that has ascended most in terms of ranking, standing seventh throughout the period analysed (1970–2019) and fourth for 2010–2019.

### 4.3. Productivity of Authors, Institutions and Countries

Table 5 presents the main variables of the articles of the 10 most prolific authors on USR during the period 1970–2019.

The authors who have published the most articles on the subject are Stavnezer (from the University of Massachusetts Medical School, USA), Luo (Huazhong University of Science and Technology, China), and Lanero (University of Leon, Spain), with four articles each.

The author with the highest number of USR citations is the Australian, Brammer, with a total of 387, which also places him as the author with the highest average number of citations per article, with 129, followed by the Americans, Stavnezer, with 302 citations and an average of 75.50 citations per article, and then Schrader, with 258 and 86, respectively. In addition, Stavnezer has the highest h-index, 4.

Of the 10 most prolific authors in the publication of articles on this research subject, four are of Spanish origin, from the University of Granada (Lanero, Garde Sánchez, Rodríguez Bolívar, and López Hernández), and three American (Stavnezer, Schrader and Hemphill).

**Figure 5:**
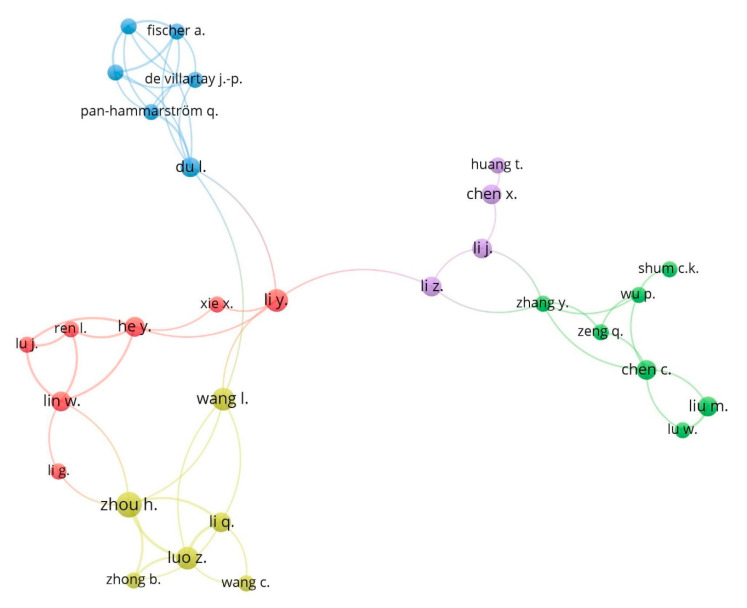
Network of cooperation between authors based on co-authorship (1970–2019).

Figure 5 shows the collaboration map among the leading authors in this research topic, based on co-authorship analysis. The different colours represent the different clusters formed by the working groups in the production of articles, and the size of the circle depends on the number of articles of each author. The main authors are grouped into 5 clusters. Cluster 1 (blue) presents the collaboration between Fischer, De Villartay, Du, and Pan-Hammarström. Meanwhile, cluster 2 (red) consists of Li, Xie, He, Ren, Lu, Lin, and Li. In the third cluster (yellow colour) groups Wang, Zhou, Li, Luo, Wang and Zhong. E. In the fourth cluster (violet), Huang, Chen, Li, and Li. Finally, the fifth cluster (green) consists of the authors Shum, Wu, Zhang, Zeng, Chen, Liu, and Lu.

Table 6 presents the 10 most productive institutions in the publication of USR-related articles. By country, the United States, with four institutions, is the first in this ranking (University of Massachusetts Medical School, Pennsylvania State University, The Ohio State University and Loyola University of Chicago). The USA is followed by China, with two institutions (Wuhan University and Chinese Academy of Sciences), and Spain, also with two (University of Cadiz and University Jaume I). Among these institutions, Wuhan University ranks first, with 10 articles published; while the York University (Canada) has the most citations, with 432, and an average of 118.17 cites per article. This institution, along with the University of Massachusetts Medical School, presents the highest h-index (5) in the Table.

The lack of international co-authorship of Spanish institutions is interesting. By contrast, 50% and 83.3%, respectively, of the articles on the economic and environmental impacts of USR from the Chinese Academy of Sciences and York University are internationally co-authored, with the same number of articles as the Spanish, University of Cadiz (6). In addition, York University shows its international credentials with the highest average number of citations per article published with international collaboration (141.60). It is also of note that the Loyola University of Chicago, despite international collaboration in 20% of their articles, have no citations in these publications.

In this sense, efforts being made by HEIs in recent years in relation to educational activity for sustainable development, are relevant, because, as UNESCO points out, education is one of the most effective tools to stimulate the changes necessary to achieve sustainable development [104,105]. For example, the UNIBILITY project of the Postgraduate Centre of the University of Vienna aims to strengthen the relationship between universities and their local communities through USR activities. It includes developing strategies on how universities can increase their social responsibility at student and research level, to USR service learning projects that affect the local community in the fields of environmental, social or economic research, and develop a USR curriculum to train university management and students [106].

Table 7 lists the main variables of the countries with the highest scientific output on the economic and environmental impacts of USR during the period 1970–2019. Heading the list is the United States, with a total of 212 articles and with the highest total number of citations (4550), meaning an average of 21.46 citations per article on the research subject, which also represents the second highest average of cites, after Canada (36.58). The United States also has the highest h-index, with 34 in published articles on USR. Following the United States as regard the highest number of articles is the United Kingdom with 87 articles, which also has the second highest number of cites (1640) and h-index (20). This peculiarity reveals the interest of American and English publications in USR [107,108].

The above data refer to the ranking of the most productive countries in the publication of articles on USR for the whole the period analysed, underlining its research potential, with 171 articles in the last period alone (2010–2019). During this last decade, China has moved into second place, with 82 articles published, replacing the United Kingdom, which held this position in the previous two decades (1990–1999, 2000–2009). These countries (United States, China, and the United Kingdom), together with Spain and Canada, are the main promoters of USR research globally, since, they have been responsible for publishing 54.94% of the world total.

The remaining five countries (Australia, Germany, India, Italy, and Japan) have published fewer articles. Of this group, Germany, with the fourth largest number of citations (987) in 27 published articles, presents the third best h-index (15) in articles on USR, and has held eighth position for the last two decades studied.

Table 8 shows the main variables related with international collaboration between countries in the publication of articles on USR. The countries with the highest percentage of international collaboration are Canada, with 65.6% of articles written in collaboration (15 articles), followed by Italy, with 60.9% (11), Australia, with 57.1% (14), Germany, with 44.4% (10), and Japan, with 42.9% (12). Spain is the country with the lowest percentage of international collaborators: 27.4% (in 11 articles).

In seven of the countries in Table 8 (the United Kingdom, China, Canada, Germany, India, Italy, and Japan), it is of note that the number of citations of articles with international co-authorship is greater than those presented by them without such collaboration.

**Figure 6:**
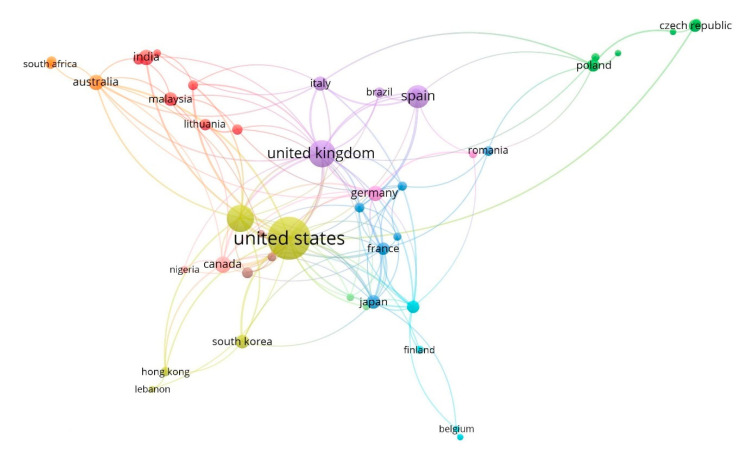
Network of cooperation between countries based on co-authorship (1970–2019).

Figure 6 shows a collaboration map between major countries based on the co-authorship of their authors. The different colours represent the different clusters formed by the groups of countries, and the size of the circle depends on the number of articles per country. In this context, the greater the circle of each country, the greater the number of articles whose authorship it represents. Countries are grouped into 11 clusters based on co-authorship analysis.

Thereby, cluster 1, the largest, includes 7 countries: India, Indonesia, Iran, Lithuania, Malaysia, Pakistan, and United Arab Emirates. Group 2 consists of Croatia, Czech Republic, Poland, Russian Federation, Slovakia, and Ukraine. Cluster 3 is led by Japan, and includes France, Hungary, Romania, Sweden, and Turkey. Cluster 4 is headed by the United States and includes China, Hong Kong, Lebanon, and South Korea. The fifth cluster, led by the United Kingdom, includes Brazil, Italy, Portugal, and Spain. Cluster 6 consists of Belgium, Finland, Netherlands, and Saudi Arabia. Group 7 consists of Australia, South Africa, and Thailand. Cluster 8 consists of Denmark, Switzerland, and Taiwan. Cluster 9 consists of Austria and Germany. Cluster 10 is made up of Canada and Algeria. Finally, cluster 11 contains Norway and Singapore.

### 4.4. Keyword Analysis

Table 9 lists the 20 most used keywords in the 870 articles published worldwide on the scope of USR during the period 1970–2019. The relationship for the entire period is shown, as well as for the ten-year sub-periods into which the 50-year period is divided.

The five most common keywords used during the period analysed (1970–2019) are sustainability (in 66 articles), priority journal (45), sustainable development (43), controlled study (36), and corporate strategy (28). As regards the first term, Sustainability, in the last period (2010–2019), co-occurrence is in 92.4% of the published articles on USR. Moreover, the literature review of the term Sustainability suggests that word is used as a synonym, or an equivalent term, of Sustainable Development [109,110].

Of the top 20 keywords, six have started to be associated with this field of research during the last period examined (2010–2019). These are stakeholders (position 9), students (12), financial performance (15), innovation (18), management (19), and perception (20).

**Figure 7:**
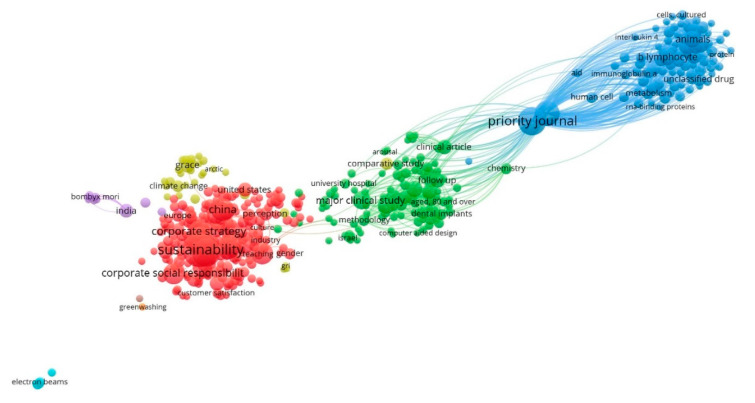
Keywords network based on co-occurrence (1970–2019).

Figure 7 represents the network map for the keywords in USR research articles in the period 1970–2019. The colour of the nodes is used to differentiate the different clusters according to the number of co-occurrences, and their size varies depending on the number of repeats. Hence, the research lines developed by different communities have been detected. Six main research lines are distinguished, which are grouped under the terms “sustainability”, “governance approach”, “greenwashing”, “management education”, “transparency”, and “environmental management”.

These lines group all terms related to the economic and environmental impacts of USR globally. Hence, the “sustainability” line includes aspects related to sustainable development in a global context of HEIs [111]. The “governance approach” line includes the study of structures and processes for the direction and control of organizations, in this field of research, focusing on HEIs [112]. The “greenwashing” line of research is a marketing concept that encompasses the bad practices of certain organizations when they submit a falsely environmentally friendly plan or proposal [113,114]. The “management education” line refers to the process of partnering people to achieve goals using resources efficiently and effectively following ethical guidelines, striving to create integrity and sustainable organizations by caring for their communities as much as possible [115]. The “transparency” line examines the demands of stakeholders on HEIs in relation to the results and impacts of HE. In consequence, transparency concerning the activities of universities has become a primary objective of management in HE [116]. Finally, the “environmental management” line refers to management and the active role of the organization related to sustainable development, that is, the triple bottom line approach [117] and the triple helix model university [118]. These keywords are related to the definitions, policies, projects, regulations, and normative practices expressed in Table 2 of Section 2.4. 

The development and evolution of this field involves new concepts and study arguments at a global level, such as “corporate citizenship and higher education behavior”, “green university”, “green campus”, “sustainable university”, “green corridors”, “circular community”, “reducing private car use”, “networked governance”, “inclusive university”, “responsibility for health”, “work-life balance”, “physical space”, “healthy lifestyle”, “healthy campus”, “eco inclusion”, or “inclusive education management”.

For all these reasons, obtaining a quality education is the basis for improving people′s lives, as well as contributing positively to sustainable development. In this sense, progress has been made in terms of access to education at all levels and in enrolment rates in universities, mainly for women and girls. On the other hand, basic literacy has increased substantially, although greater efforts are needed to achieve the goals of universal education and achieve equality for women in the University and their insertion in technical careers.

Hence, the quality management of HEIs are aligned with sustainability and with the development of an egalitarian society, with the purpose of SDG 4 guaranteeing inclusive, equitable and quality education, and promoting lifelong learning opportunities [78,82].

In recent years, HEIs are undergoing changes at a global level, albeit in a heterogeneous way. It is key to admit that there is a disparity in performance, but also in the resources, capacities, and scope in which these institutions carry out their activity. The modernization of the universities has meant that their focus is turned from the academic and research perspective, and pay attention to the stakeholders, with special reference to the characteristics of their immediate geographical context that marks the environment of competition and the available potential markets. The organizational heterogeneity of the different HEIs has a direct impact on the implications in the decisions that are related to the economic, social, and environmental aspects.

### 4.5. Future Studies

**Figure 8:**
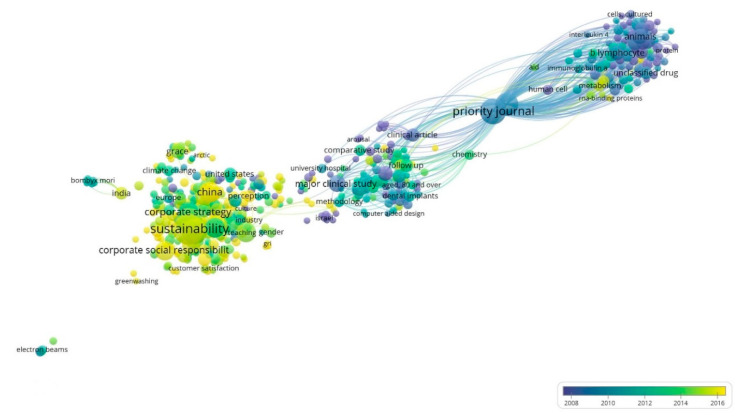
Evolution of keywords network based on co-occurrence (1970–2019).

Figure 8 shows the mature of each keyword community by differentiating the period in which they have been studied. Thus, the red cluster belongs to the period 2014–2016, the most recent, while blue and green clusters have a large number of keywords more closely linked to the field of research. This graph identifies the importance of keywords according to the time in which they became associated with USR, because most pioneers will have greater influence and will become a point of reference for those that have subsequently emerged. However, the existence of six distinct clusters illustrates how USR has incorporated numerous subjects of study into research activity.

The evolution of research on this topic encourages the appearance of different projects at the international level, with the aim of considering a positive environmental impact, a sustainable and responsible consumption, to offer a more sustainable future in relation to the SDGs.

In the context of both corporate sustainability, SDGs and responsible consumption, initiatives emerge, such as, the EuroHealthNet research platform promotes evidence-based approaches to health, equity and well-being, through collaboration between decision makers and researchers and their results. In this regard, it provides an evidence base for policies and practices that address health inequalities and environmental threats to health [119]. Likewise, EuroHealhNet collaborates with CHAIN (Center for Global Health Inequalities Research) in CHAIN′s Global Health Inequalities project (2019–2025), which brings together researchers at the international level and different research disciplines in order to advance experimental ideas social, laboratory and natural aspects of the causal mechanisms that link socioeconomic status and health [120].

These initiatives promote sustainable consumption to improve sustainable performance, in addition to meeting the needs of consumers, and increasing the quality of life of citizens. It is about improving, through public or private initiatives, both the efficiency of resources, as well as increasing the use of renewable energy, reducing carbon emissions, and minimizing waste [121,122,123,124].

Future studies on this topic should focus on new research lines that will determine, among others, whether the ethical code of a given university reflects its commitment to sustainable development; or, also, to evaluate, as a sustainability challenge, the participation of HE in business ethics.

Furthermore, on the other hand, the research must identify what actions are necessary for a more responsible future, related to the economy, demography, sustainability, health, well-being, social inequalities, equity, and the environment.

Even, it should be analysed how the promotion of educational practices on social responsibility and focused on the development of the consciousness of millennial students, will have a positive effect on responsible consumption. Green marketing, from both educational institutions and their providers, must be extensively studied to highlight these fraudulent practices, which undermine the image of HEIs.

It would also be necessary, on the other hand, to develop academic and scientific contributions that establish the dimensions that contribute to the strengthening of the USR from the social, economic, healthy, welfare and sustainable points of view.

These new thematic axes should also create models that measure the satisfaction of interested parties with the practical aspects of the USR, institutionalize the link between USR and learning services; evaluate the impact of strategies that develop partnerships between HEIs and other institutions or organizations with respect to achieving the objective of sustainable development, and analyse the links between the responsible and sustainable consumption of universities and their short-term financial, economic, and sustainable impacts.

In short, research at the global level, both from the initiative of the individual researcher and from joint projects, should develop studies that extol the sustainable consumption of HEIs in their USR actions, from the consumption of products and services that minimize the environmental impact, maximize the economic impact, and satisfy the needs of the societies to which they are linked, for both present and future generations.

## 5. Conclusions

The objective of this study was to analyse the scientific production of the economic and environmental impacts of USR in the last 50 years through a bibliometric analysis of 870 articles obtained from the Scopus database. The findings made it possible to identify the main drivers, potential tendencies, and certain gaps in critical knowledge. It is concluded that the USR, as a mechanism that allows the dissemination of HEI values and so ensures their economic, environmental, and social sustainability, is committed to the demands of its various interest groups. Thereby, the thematic areas, journals, authors, institutions, and countries involved USR publications have been identified.

The number of scientific papers published per year during the period 1970–2019 has increased, especially in the last decade, when 743 articles were published, representing 85.70% of total contributions to this research topic.

The thematic area of business, management, and accounting is the most common, since it groups 27% of the articles, followed by Social Sciences with 25%, and Environmental Science with 14%. Furthermore, regulatory developments have influenced the development of articles related to the business, management, and accounting and social sciences subject areas.

The most productive journal on the scope of USR worldwide has been the British journal, Business and Society Review, with 50 publications, representing 5.75% of the total number of articles published in the study period. Of the journals that contribute to this topic, 45% are positioned in the first quartile of the Scopus database. The American journal, California Management Review has the highest number of citations (2,098), the highest average number of citations per article (87.82) and the highest h-index for published articles in this subject area (15).

The authors who have published the most on the subject are Stavnezer (USA), Luo (China), and Lanero (Spain), with four articles each. Brammer, an Australian from Macquarie University, is the author with the highest number of citations (387), and the best average citations per article (129). Stavnezer is also the author with the highest h-index for contributions on USR (4).

The most productive institution in this research area is Wuhan University, China, with 10 articles. The Canadian York University has the highest number of citations (709), the best average citations per article (118.17), and it is the institution with the most international collaborations in its work on the USR (83.3%). Among the institutions that have contributed the most to the subject but with no international collaboration in their articles during the period studied are Cadiz University and Jaume I University (Spain) and Loyola University of Chicago (USA).

The main countries that have contributed to this area of research, in terms of the number of articles and international collaboration, during the period 1970–2019, are, in order, the United States, the United Kingdom, China, Spain, and Canada. The United States presents the largest number of published articles (212) and citations (4550), in addition to the highest h-index, with 34.

Future research lines on this topic will focus on (i) determining whether the ethical codes of a given university reflect its commitment to sustainable development, (ii) assessing the involvement of HE in business ethics like a challenge of sustainability, (iii) identifying what actions are needed for a healthier future, related to the economy, demography, sustainability, health, wellbeing, social inequalities, equity, and the environment, and (iv) promoting educational practices focused on the development of millennial students′ awareness of social responsibility and investment.

Among the main future research lines to be pursued are, principally, those that must (i) determine whether the ethical code of a given university reflects its commitment to sustainable development; (ii) evaluate the participation of HE in business ethics; (iii) identify the actions necessary for a more responsible future, (iv) analyse how the promotion of educational practices on social responsibility and focused on the development of the consciousness of millennial students will have a positive effect on responsible consumption; (v) study green marketing, both from educational institutions and from their suppliers; or (vi) analyse the links between the responsible and sustainable consumption of universities and their short-term financial, economic, and sustainable impacts.

This study supposes an analysis of the scientific production and the actors that stimulate the investigation of the socio-economic and environmental dimensions on University Social Responsibility during the period 1970–2019, as well as the identification of the lines of investigation and their evolution and transformation. Innovation in this research field has been identified based on the morphology of the groups of authors, institutions, countries, and keywords, and the intensity of the relationships that develop in them. The results obtained are a complement to the knowledge of the evolution of the socio-economic and environmental dimensions on USR and allow to establish the relationship between science and technology, and to inform the decision-making process.

Likewise, this research is of interest since it makes known the importance of USR in training responsible citizens with their environment, generators of creative ideas and committed to help solve social and environmental problems. This study contributes to disseminating the need for universities to make available to students, not only knowledge but also the possibility of developing competencies and skills, which give them the opportunity to transform their participation in social action initiatives.

With a triple-pronged approach, the USR allows the implementation of progress policies and actions that lead towards sustainability. In this way, the research we carry out fosters a greater knowledge of the tools for more sustainable progress in our society. In this sense, the investigation and dissemination of these terms allows the promotion of initiatives that promote the ethical conduct of the citizen towards herself/himself and with her/his environment, and goes far beyond compliance with legal obligations, but seeks to raise awareness and respond to forge responsible students with their environment and with humanity.

Finally, it should be noted that research on the economic and environmental impacts of USR worldwide has followed an upward trend of exponential growth, especially in the last decade studied, namely 2010–2019.

## Figures and Tables

**Table 1:** Main literature revised of the research topic.

Reference	Author(s)	Year	Journal
Gender-specific health behaviors of German university students predict the interest in campus health promotion [35]	Stock, C.	2007	Health Promotion International
Effect of business education on women and men students’ attitudes on corporate responsibility in society [36]	Lämsä, A.-M., Vehkaperä, M., Puttonen, T., Pesonen, H.-L.	2007	Journal of Business Ethics
Responsabilidad Social Universitaria: una nueva filosofía de gestión ética e inteligente para las universidades [37]	Vallaeys, F.	2008	Educación superior y sociedad
Corporate social responsibility in higher education [38]	Brown, E., Cloke, J.	2009	ACME: An International Journal for Critical Geographies
Performance indicators and corporate social responsibility: Evidence from Portuguese higher education institutions [39]	David, F., Abreu, R., Carreira, F., Gonçalves, S.	2010	International Journal of Banking, Accounting and Finance
Can a university act as a corporate social responsibility (CSR) driver? An analysis [40]	Ahmad, J.	2012	Social Responsibility Journal
Online disclosure of university social responsibility: A comparative study of public and private US universities [41]	Garde Sánchez, R., Rodríguez Bolívar, M.P., López-Hernández, A.M.	2013	Environmental Education Research
What is the social responsibility of a Higher Education Institution (HEI) toward sustainable energy supply: Advocacy on responsible energy utilization [42]	Florida, J.S., Paladan, N.N.	2014	International Journal of Sustainability Education
Higher education institutions and social performance: Evidence from public and private universities [43]	Othman, R., Othman, R.	2014	International Journal of Business and Society
University social responsibility (USR): Identifying an ethical foundation within higher education institutions [44]	Chen, S.-H.A., Nasongkhla, J., Donaldson, J.A.	2015	Turkish Online Journal of Educational Technology
University–community engagement: Case study of university social responsibility [45]	Chile, L.M., Black, X.M.	2015	Education, Citizenship and Social Justice
The effect of CSR on learning and growth perspective in Private Universities [46]	Al-Hosaini, F.F., Sofian, S.	2015	Mediterranean Journal of Social Sciences
University social responsibility as antecedent of students’ satisfaction [47]	Vázquez, J.L., Aza, C.L., Lanero, A.	2016	International Review on Public and Nonprofit Marketing
University social responsibility and brand image of private universities in Bangkok [48]	Plungpongpan, J., Tiangsoongnern, L., Speece, M.	2016	International Journal of Educational Management
Examining obligations to society for QS stars best ranked universities in social responsibility [49]	Păunescu, C., DrăganGilmeanu, D., Găucă, O.	2017	Management and Marketing

**Table 2:** University Social Responsibility: definitions, policies, projects, regulations, normative and practices.

Year	International Organization	Resource/Initiative
1998	United Nations Educational, Scientific and Cultural Organization (UNESCO)	World Declaration on Higher Education in the 21st Century
2000	United Nations	Millennium Development Goals (MDGs)
2006	Council of Europe	Declaration of Responsibility for Higher Education for a Democratic Culture: Citizenship, Human Rights and Sustainability
2008	International Organization for Standardization (ISO)	ISO 9001: Quality management
2009	United Nations Educational, Scientific and Cultural Organization (UNESCO)	World Conference on Higher Education: The new dynamics of higher education and research for social change and development
2010	International Organization for Standardization (ISO)	ISO 26000: International Organization for Standardization’s Social Responsibility Guide
2011	European Commission	Renewed EU strategy 2011-14 on corporate social responsibility
2015	United Nations	The 2030 Agenda for Sustainable Development – Sustainable Development Goals
2016	European Commission	INHERIT (INter-sectoral Health and Environment Research for InnovaTion)
2017	Global University Network for Innovation (GUNi)	Guidelines for universities engaging in social responsibility
2017	Global University Network for Innovation (GUNi)	Higher Education in the World 6. Towards a Socially Responsible University: Balancing the Global with the Local” (HEIW6)
2017	Forética	SGE Standard 21: Ethical and Socially Responsible Management System

**Table 3:** Major characteristics of the articles (1970–2019).

Period	A	AU	C	TC	TC/A	J
1970–1979	7	11	2	813	116.1	2
1980–1989	13	19	2	904	69.5	5
1990–1999	16	45	7	579	36.2	16
2000–2009	91	259	31	3457	38.0	34
2010–2019	743	2001	86	5737	7.7	397

A: number of articles; AU: number of authors; C: number of countries; TC: number of citations in total articles; TC/A: number of citations by article; J: number of journals.

**Table 4:** The most productive journals in number of articles (1970–2019).

Journal	R(A)	TC	TC/A	H(A)	H(J)	SJR(Q)	C	R (A)				
								1970–79	1980–89	1990–99	2000–09	2010–19
Business and Society Review	50	266	5.32	10	17	0.322(Q2)	UK	0	0	0	6(2)	1(48)
Business Horizons	24	960	40.00	9	67	1.296(Q1)	Netherlands	0	0	0	3(6)	2(18)
California Management Review	24	2098	87.42	15	118	2.658(Q1)	USA	1(6)	1(8)	0	0	9(10)
Journal of Business Ethics	19	466	24.53	11	147	1.860(Q1)	Netherlands	0	0	12(1)	2(7)	6(11)
Polish Journal of Management Studies	14	48	3.43	4	11	0.353(Q2)	Poland	0	0	0	0	3(14)
Socio Economic Review	13	958	73.69	11	45	2.401(Q1)	UK	0	0	0	14(2)	7(11)
Sustainability Switzerland	13	36	2.77	4	53	0.549(Q2)	Switzerland	0	0	0	0	4(13)
Asia Pacific Journal of Accounting and Economics	12	56	4.67	4	10	0.209(Q3)	UK	0	0	0	0	5(12)
Journal of Experimental Medicine	11	723	65.73	11	411	7.941(Q1)	USA	0	0	0	1(11)	0
Voluntas	11	132	12.00	7	41	0.610(Q1)	USA	0	0	0	0	8(11)
Social Responsibility Journal	10	92	9.20	5	23	0.432(Q2)	UK	0	0	0	54(1)	13(9)
Transformations in Business and Economics	10	19	1.90	3	15	0.284(Q2)	Lithuania	0	0	0	0	10(10)
E a M: Ekonomie a Management	9	30	3.33	3	18	0.318(Q2)	Czech Republic	0	0	0	0	11(9)
Journal of Industrial Ecology	9	175	19.44	6	85	1.486(Q1)	USA	0	0	0	11(2)	17(7)
Journal of Management and Organization	9	95	10.56	5	27	0.385(Q2)	UK	0	0	0	0	12(9)
Environmental Engineering and Management Journal	8	31	3.88	3	31	0.345(Q3)	Romania	0	0	0	29(1)	15(7)
Journal of Cleaner Production	8	169	21.13	5	150	1.620(Q1)	Netherlands	0	0	0	0	14(8)
Nucleic Acids Research	8	175	21.88	8	452	8.636(Q1)	UK	0	0	15(1)	0	19(7)
Community Development Journal	7	157	22.43	7	37	0.462(Q2)	UK	0	0	0	8(2)	23(5)
Journal of Business Economics and Management	7	31	4.43	2	30	0.389(Q2)	Lithuania	0	0	0	0	16(7)

A: number of articles; TC: number of citations for all articles; TC/A: number of citations by article; H(A): h-index in articles; H(J): h-index in journal; SJR: Scimago Journal Rank (Quartile); R: rank position by number of articles.

**Table 5:** The most productive authors in number of articles (1970–2019).

Author	A	TC	TC/A	Institution	C	1st A	Last A	H-Index
Stavnezer, J.	4	302	75.50	University of Massachusetts Medical School	USA	2005	2007	4
Luo, Z.	4	23	5.75	Huazhong University of Science and Technology	China	2015	2019	3
Lanero, A.	4	8	2.00	University of Leon	Spain	2012	2013	2
Brammer, S.	3	387	129.00	Macquarie University	Australia	2005	2012	3
Schrader, C.E.	3	258	86.00	University of Massachusetts Medical School	USA	2005	2007	3
Solomon, Z.	3	69	23.00	Bob Shapell School of Social Work	Israel	1987	2011	3
Garde Sánchez, R.	3	57	19.00	University of Granada	Spain	2013	2015	3
Rodríguez Bolívar, M.P.	3	57	19.00	University of Granada	Spain	2013	2015	3
López Hernández, A.M.	3	38	12.67	University of Granada	Spain	2013	2015	3
Hemphill, T.A.	3	27	9.00	University of Michigan-Flint	USA	2010	2018	2

A: number of articles; TC: number of citations for all articles; TC/A: number of citations by article; 1st A: First article; Last A: Last article; h-index: Hirsch index in research topic.

**Table 6:** The most productive institutions in number of articles (1970–2019).

Institution	Country	A	TC	TC/A	H-Index	IC (%)	TCIC	TCNIC
Wuhan University	China	10	48	4.80	4	30.0%	10.00	2.57
Universidad de Cadiz	Spain	6	38	6.33	3	0.0%	0.00	6.33
Chinese Academy of Sciences	China	6	37	6.17	4	50.0%	7.33	5.00
York University	Canada	6	709	118.17	5	83.3%	141.60	1.00
University of Massachusetts Medical School	USA	5	314	62.80	5	40.0%	60.00	64.67
Pennsylvania State University	USA	5	97	19.40	3	20.0%	20.00	19.25
Universiteit van Amsterdam	Netherlands	5	101	20.20	2	40.0%	48.50	1.33
Universidad Jaume I	Spain	5	23	4.60	3	0.0%	0.00	4.60
The Ohio State University	USA	5	304	60.80	3	40.0%	19.00	88.67
Loyola University of Chicago	USA	5	203	40.60	3	20.0%	0.00	50.75

A: number of articles; TC: number of citations for all articles; TC/A: number of citations by article; h-index: Hirsch index in research topic; IC: percentage of articles made with international collaboration; TCIC: number of citations by article made with international collaboration; TCNIC: number of citations by article made without international collaboration.

**Table 7:** The most productive countries in number of articles (1970–2019).

Country	A	TC	TC/A	h-Index	R (A)				
					1970–1979	1980–1989	1990–1999	2000–2009	2010–2019
USA	212	4550	21.46	34	1(7)	2(1)	1(8)	1(25)	1(171)
UK	87	1640	18.85	20	0	0	2(2)	2(17)	3(68)
China	85	388	4.56	11	0	0	0	7(3)	2(82)
Spain	62	921	14.85	11	0	0	0	6(4)	4(58)
Canada	32	1161	36.28	13	0	0	4(1)	3(6)	7(25)
Australia	28	307	10.96	9	0	0	0	16(1)	5(27)
Germany	27	987	36.56	15	0	0	0	8(3)	8(24)
India	27	81	3	6	0	0	0	0	6(27)
Italy	23	203	8.83	7	0	0	0	20(1)	9(22)
Japan	21	276	13.14	9	0	0	6(1)	5(5)	16(15)

A: number of articles; R: rank position by number of articles; TC: number of citations for all articles; TC/A: number of citations by article; h-index: Hirsch index in research topic.

**Table 8:** The most productive countries and international collaboration (1970–2019).

Country	TC	Main Collaborators Countries	IC (%)	TC/A	
				IC	NIC
USA	31	Canada, China, UK, Pakistan, South Korea	25.9%	16.00	23.38
UK	27	USA, Canada, Italy, China, Germany	36.8%	28.06	13.49
China	17	USA, UK, Australia, Canada, Germany	28.2%	9.42	2.66
Spain	11	Portugal, Italy, Brazil, Sweden, UK	27.4%	4.29	18.84
Canada	15	USA, UK, China, Hong Kong, Netherlands	65.6%	44.14	21.27
Australia	14	USA, China, Italy, UK, Albania	57.1%	8.13	14.75
Germany	10	UK, Austria, China, France, Australia	44.4%	46.67	28.47
India	5	USA, Indonesia, Papua New Guinea, United Arab Emirates, UK	22.2%	5.50	2.29
Italy	11	UK, Spain, Australia, Netherlands, Poland	60.9%	12.71	2.78
Japan	12	Sweden, USA, Belgium, Canada, China	42.9%	22.22	6.33

TC: total number of citations for all articles; A: number of articles; TC/A: number of citations by article; IC: percentage of articles made with international collaboration; NIC: number of citations by article made without international collaboration.

**Table 9:** Main keywords on USR research (1970–2019).

Keyword	1970–2019		1970–1979		1980–1989		1990–1999		2000–2009		2010–2019	
	R (A)	%	R (A)	%	R (A)	%	R (A)	%	R (A)	%	R (A)	%
Sustainability	66	7.6%	0	0.0%	0	0.0%	0	0.0%	16(5)	5.5%	1(61)	8.2%
Priority Journal	45	5.2%	0	0.0%	28(1)	7.7%	1(4)	25.0%	1(19)	20.9%	5(21)	2.8%
Sustainable Development	43	4.9%	0	0.0%	0	0.0%	0	0.0%	32(4)	4.4%	2(39)	5.2%
Controlled Study	36	4.1%	0	0.0%	0	0.0%	3(2)	12.5%	2(18)	19.8%	8(16)	2.2%
Corporate Strategy	28	3.2%	0	0.0%	0	0.0%	0	0.0%	10(5)	5.5%	4(23)	3.1%
Ethics	27	3.1%	0	0.0%	0	0.0%	66(1)	6.3%	0	0.0%	3(25)	3.4%
Corporate Governance	22	2.5%	0	0.0%	0	0.0%	0	0.0%	96(2)	2.2%	6(20)	2.7%
Major Clinical Study	19	2.2%	0	0.0%	21(1)	7.7%	2(3)	18.8%	60(3)	3.3%	10(12)	1.6%
Stakeholders	18	2.1%	0	0.0%	0	0.0%	0	0.0%	0	0.0%	7(17)	2.3%
Middle Aged	17	2.0%	0	0.0%	0	0.0%	103(1)	6.3%	28(4)	4.4%	11(12)	1.6%
Adolescent	14	1.6%	1(1)	14.3%	1(3)	23.1%	0	0.0%	0	0.0%	17(9)	1.2%
Students	13	1.5%	0	0.0%	0	0.0%	0	0.0%	0	0.0%	9(13)	1.7%
Business Ethics	12	1.4%	0	0.0%	0	0.0%	0	0.0%	0	0.0%	12(11)	1.5%
Clinical Article	12	1.4%	0	0.0%	10(1)	7.7%	29(1)	6.3%	20(4)	4.4%	50(6)	0.8%
Financial Performance	11	1.3%	0	0.0%	0	0.0%	0	0.0%	0	0.0%	13(10)	1.3%
Retrospective Studies	11	1.3%	0	0.0%	0	0.0%	143(1)	6.3%	0	0.0%	26(8)	1.1%
Human Rights	10	1.1%	0	0.0%	0	0.0%	0	0.0%	53(3)	3.3%	35(7)	0.9%
Innovation	10	1.1%	0	0.0%	0	0.0%	0	0.0%	0	0.0%	14(10)	1.3%
Management	10	1.1%	0	0.0%	0	0.0%	0	0.0%	0	0.0%	20(9)	1.2%
Perception	10	1.1%	0	0.0%	0	0.0%	0	0.0%	0	0.0%	15(10)	1.3%

R(A): rank position by number of articles; A: number of articles; %: percentage of articles in which it appears.

## References

[1] Motoyama Y., Mayer H. (2017). Revisiting the Roles of the University in Regional Economic Development: A Triangulation of Data. Growth Chang..

[2] Moggi S. (2019). Social and environmental reports at universities: A Habermasian view on their evolution. Account. Forum.

[3] Fedyunin D.V., Lochan S.A., Avtonomova S.A., Kutyrkina L.V., Mintusov I.E. (2018). The Scenario Approach to Establishing GR Communications in Higher Education Institutions. J. Adv. Res. Law Econ..

[4] Yob I.M. (2016). Cultural Perspectives on Social Responsibility in Higher Education. High. Learn. Res. Commun..

[5] Geryk M. (2011). Social Responsibility of Higher Education Institution as Manifestation of Positive Organizational Scholarship. J. Posit. Manag..

[6] Hawkins R.A. (1976). Education and Social Action: Community Service and the Curriculum in Higher Education. J. High. Educ..

[7] Hongdiyanto C. (2018). Attribute in designing entrepreneurial Learning Model in Higher Education. Int. J. Acad. Res. Bus. Soc. Sci..

[8] Jařab J. (2008). Reforming systems and institutions of higher learning. Educ. Citizsh. Soc. Justice.

[9] Predazzi E. (2012). The third mission of the university. Rend. Lincei.

[10] Levy N. (2019). Taking Responsibility for Responsibility. Public Health Ethics.

[11] Bell R., Khan M., Romeo-Velilla M., Stegeman I., Godfrey A., Taylor T., Morris G., Staatsen B., van der Vliet N., Kruize H. (2019). Ten Lessons for Good Practice for the INHERIT Triple Win: Health, Equity, and Environmental Sustainability. Int. J. Environ. Res. Public Health.

[12] Cornelius N., Wallace J., Tassabehji R. (2007). An analysis of corporate social responsibility, corporate identity and ethics teaching in business schools. J. Bus. Ethics.

[13] Sunardi S. (2019). University Social Responsibility, University Image and Hight Education Performance. Indones. Manag. Account. Res..

[14] Jeffrey S., Rosenberg S., McCabe B. (2019). Corporate social responsibility behaviors and corporate reputation. Soc. Responsib. J..

[15] Franceschini F., Galetto M., Mastrogiacomo L. (2018). ISO 9001 certification and failure risk: Any relationship?. Total Qual. Manag. Bus. Excell..

[16] Yeung S.M.C. (2018). Linking ISO 9000 (QMS), ISO 26000 (CSR) with accreditation requirements for quality indicators in higher education. Total Qual. Manag. Bus. Excell..

[17] Harjoto M.A. (2017). Corporate social responsibility and corporate fraud. Soc. Responsib. J..

[18] Choi H., Choi B., Byun J. (2018). The relationship between corporate social responsibility and earnings management: Accounting for endogeneity. Investig. Manag. Financ. Innov..

[19] Nejati M., Shafaei A., Salamzadeh Y., Daraei M. (2011). Corporate social responsibility and universities: A study of top 10 world universities’ websites. Afr. J. Bus. Manag..

[20] Tetřevová L. (2013). Modification of the Corporate Social Responsibility Concept in the context of Universities’ Social Responsibility. Acta Acad. Karviniensia.

[21] Almeida Pástor M., Arrechavaleta Guarton C.N. (2018). Responsabilidad social empresarial y sus limitaciones en el contexto académico universitario. Rev. Cuba. Educ. Super..

[22] López Cambronero M. (2003). Cultura, ciencia y Universidad: Los análisis y propuestas de José Ortega y Gasset. Daimon Rev. Int. Filos..

[23] Kerr C. (1991). Ortega y Gasset for the 21st Century. Society.

[24] Allen Gaw E. (1930). Social Education. J. High. Educ..

[25] Sheehan J. (1997). Social Demand versus Political Economy in Higher Education at the Turn of the Century Higher Education Financing: Policy Options. High. Educ. Eur..

[26] Auzan A. (2013). University Mission: An Economist’s Perspective. Educ. Stud..

[27] Bowen H.R. (1984). Graduate education and social responsibility. New Dir. High. Educ..

[28] Sepasi S., Braendle U., Rahdari A.H. (2019). Comprehensive sustainability reporting in higher education institutions. Soc. Responsib. J..

[29] Gaddy S. (2018). Help your students understand access at your institution. Disabil. Compliance High. Educ..

[30] Heath P. (2000). Education as Citizenship: Appropriating a new social space. High. Educ. Res. Dev..

[31] Leydesdorff L. (2012). The triple helix, quadruple helix…, and an N-tuple of helices: Explanatory models for analyzing the knowledge-based economy?. J. Knowl. Econ..

[32] Etzkowitz H., Leydesdorff L. (1998). A Triple Helix of University—Industry—Government Relations. Ind. High. Educ..

[33] Etzkowitz H. (2003). Innovation in innovation: The triple helix of university-industry-government relations. Soc. Sci. Inf..

[34] Etzkowitz H., Zhou C. (2006). Triple Helix twins: Innovation and sustainability. Sci. Public Policy.

[35] Stock C. (2001). Gender-specific health behaviors of German university students predict the interest in campus health promotion. Health Promot. Int..

[36] Lämsä A.-M., Vehkaperä M., Puttonen T., Pesonen H.-L. (2007). Effect of Business Education on Women and Men Students’ Attitudes on Corporate Responsibility in Society. J. Bus. Ethics.

[37] Vallaeys F. (2008). Responsabilidad Social Universitaria: Una nueva filosofía de gestión ética e inteligente para las universidades. Educ. Super. Soc..

[38] Brown E., Cloke J. (2009). Corporate social responsibility in higher education. ACME Int. e-J. Crit. Geogr..

[39] David F., Abreu R., Carreira F., Goncalves S. (2010). Performance indicators and corporate social responsibility: Evidence from Portuguese higher education institutions. Int. J. Bank. Account. Financ..

[40] Ahmad J. (2012). Can a university act as a corporate social responsibility (CSR) driver? An analysis. Soc. Responsib. J..

[41] Garde Sánchez R., Rodríguez Bolívar M.P., López-Hernández A.M. (2013). Online disclosure of university social responsibility: A comparative study of public and private US universities. Environ. Educ. Res..

[42] Florida J., Paladan N. (2014). What is the Social Responsibility of a Higher Education Institution (HEI) toward Sustainable Energy Supply: Advocacy on Responsible Energy Utilization. Int. J. Sustain. Educ..

[43] Othman R., Othman R. (2014). Higher Education Institutions and Social Performance: Evidence from Public and Private Universities. Int. J. Bus. Soc..

[44] Chen S.H., Nasongkhla J., Donaldson J.A. (2015). University Social Responsibility (USR): Identifying an Ethical Foundation within Higher Education Institutions. Turk. Online J. Educ. Technol.-TOJET.

[45] Chile L.M., Black X.M. (2015). University–community engagement: Case study of university social responsibility. Educ. Citizsh. Soc. Justice.

[46] Al-Hosaini F.F., Sofian S. (2015). The Effect of CSR on Learning and Growth Perspective in Private Universities. Mediterr. J. Soc. Sci..

[47] Vázquez J.L., Aza C.L., Lanero A. (2016). University social responsibility as antecedent of students’ satisfaction. Int. Rev. Public Nonprofit Mark..

[48] Plungpongpan J., Tiangsoongnern L., Speece M. (2016). University social responsibility and brand image of private universities in Bangkok. Int. J. Educ. Manag..

[49] Păunescu C., Drăgan D., Găucă O. (2017). Examining obligations to society for QS Stars best ranked universities in social responsibility. Manag. Mark..

[50] Gerholz K.-H., Heinemann S. (2015). CSR—A New Challenge for Universities? A Theoretical and Empirical Analysis of German Universities. New Perspect. Corp. Soc. Responsib..

[51] Maidin F.N., Izhar T.A. (2018). Framework Based Knowledge Sharing Factor in Higher Institution. Int. J. Acad. Res. Bus. Soc. Sci..

[52] Larrán-Jorge M., Andrades-Peña F.J. (2015). Análisis de la responsabilidad social universitaria desde diferentes enfoques teóricos. Rev. Iberoam. Educ. Super..

[53] Tetřevová L., Sabolova V. (2010). University stakeholder management and university social responsibility. WSEAS Trans. Adv. Eng. Educ..

[54] Peyman N., Sedghi F. (2019). Health Promoting University; Necessity and Framework. J. Educ. Community Health.

[55] Freeman R.E. (1994). The Politics of Stakeholder Theory: Some Future Directions. Bus. Ethics Q..

[56] Carson T.L. (1993). Does the stakeholder theory constitute a new kind of theory of social responsibility?. Bus. Ethics Q..

[57] McWilliams A., Siegel D. (2001). Corporate social responsibility: A theory of the firm perspective. Acad. Manag. Rev..

[58] Phillips R., Freeman R.E., Wicks A.C. (2003). What stakeholder theory is not. Bus. Ethics Q..

[59] Plaza-Úbeda J.A., De Burgos-Jiménez J., Belmonte-Ureña L.J. (2011). Stakeholders. Environmental management and performance: An integrated approach. Cuad. Econ. Dir. Empresa.

[60] Román-Sánchez I.M., Aznar-Sánchez J.A., Belmonte-Ureña L.J. (2015). Heterogeneity of the environmental regulation of industrial wastewater: European wineries. Water Sci. Technol..

[61] Latif K.F. (2018). The development and validation of stakeholder-based scale for measuring university social responsibility (USR). Soc. Indic. Res..

[62] Carroll A.B. (1991). The pyramid of corporate social responsibility: Toward the moral management of organizational stakeholders. Bus. Horiz..

[63] Carroll A.B., Shabana K.M. (2010). The business case for corporate social responsibility: A review of concepts, research and practice. Int. J. Manag. Rev..

[64] Friedman M., Zimmerli W.C., Holzinger M., Richter K. (2007). The Social Responsibility of Business Is to Increase Its Profits. Corporate Ethics and Corporate Governance.

[65] Dahlsrud A. (2008). How corporate social responsibility is defined: An analysis of 37 definitions. Corp. Soc. Responsib. Environ. Manag..

[66] Lee E.-M., Lee H.J., Pae J.-H., Park S.-Y. (2016). The important role of corporate social responsibility capabilities in improving sustainable competitive advantage. Soc. Responsib. J..

[67] Przychodzeń W., Przychodzeń J. (2014). Corporate Social Responsibility for Sustainability. Manag. Bus. Adm. Cent. Eur..

[68] DiSegni D.M., Huly M., Akron S. (2015). Corporate social responsibility, environmental leadership and financial performance. Soc. Responsib. J..

[69] McDonald S. (2014). Social responsibility clusters arising from social partnerships. Soc. Responsib. J..

[70] WCED, World Commission on Environment and Development (1987). Our Common Future.

[71] Jiang H., Shao Q., Liou J.J.H., Shao T., Shi X. (2019). Improving the Sustainability of Open Government Data. Sustainability.

[72] Elkington J. (1998). Partnerships from cannibals with forks: The triple bottom line of 21st-century business. Environ. Qual. Manag..

[73] Alhaddi H. (2015). Triple bottom line and sustainability: A literature review. Bus. Manag. Stud..

[74] Klumpp M. (2018). How to Achieve Supply Chain Sustainability Efficiently? Taming the Triple Bottom Line Split Business Cycle. Sustainability.

[75] Dyck B., Walker K., Caza A. (2019). Antecedents of sustainable organizing: A look at the relationship between organizational culture and the triple bottom line. J. Clean. Prod..

[76] Strachan G. (2018). Can Education for Sustainable Development Change Entrepreneurship Education to Deliver a Sustainable Future?. Discourse Commun. Sustain. Educ..

[77] Huckle J., Wals A.E.J. (2015). The UN Decade of Education for Sustainable Development: Business as usual in the end. Environ. Educ. Res..

[78] Von Geibler J., Piwowar J., Greven A. (2019). The SDG-Check: Guiding Open Innovation towards Sustainable Development Goals. Technol. Innov. Manag. Rev..

[79] Pompeu R., Marques C., Braga V., Karataş-Ozkan M., Nicolopoulou K., Özbilgin M.F. (2014). The influence of university social responsibility on local development and human capital. Corporate Social Responsibility and Human Resource Management: A Diversity Perspective.

[80] Rotengruber P. (2018). Words and deeds. Corporate Social Responsibility in higher education institutions. Annales. Etyka w Życiu Gospod..

[81] Kostiuk T.O. (2018). University social responsibility as a part of state corporate responsibility. Public Adm. Cust. Adm..

[82] Odongo N.H., Wang D. (2018). Corporate responsibility, ethics and accountability. Soc. Responsib. J..

[83] Kouatli I. (2019). The contemporary definition of university social responsibility with quantifiable sustainability. Soc. Responsib. J..

[84] Pernía J.C. (2018). Visión de la Gestión en la Gerencia de la Responsabilidad Social Universitaria. Rev. Sci..

[85] Parsons A. Literature Review on Social Responsibility in Higher Education; Occasional Paper Number 2; UNESCO Chair for Community Based Research and Social Responsibility in Higher Education: 2014. http://unescochair-cbrsr.org/unesco/pdf/Occaisional_paper.pdf.

[86] Ab Wahid R. (2019). Sustaining ISO 9001-based QMS in higher education: A reality?. TQM J..

[87] Caret R.L. (2019). Social Responsibility and Civic Readiness as Critical Higher Education Outcomes. Metrop. Univ..

[88] Wided R. (2019). University Social Responsibility and Sustainable Development Awareness: The Mediating Effect of Corporate Social Responsibility Case of Qassim University. J. Soc. Sci. Stud..

[89] Bolton D., Landells T. (2019). Marketised Higher Education: Implications for Corporate Social Responsibility and Social Licence to Operate. J. Sustain. Res..

[90] INHERIT (INter-sectoral Health and Environment Research for InnovaTion). https://www.inherit.eu/.

[91] Vlachý J. (1979). Quotations and scientometrics. Scientometrics.

[92] Sen B.K., Shailendra K. (1992). Evaluation of recent scientific research output by a bibliometric method. Scientometrics.

[93] Belmonte-Urenã L.J., Garrido-Cardenas J.A., Camacho-Ferre F. (2020). Analysis of world research on grafting in horticultural plants. HortScience.

[94] Abad-Segura E., González-Zamar M.-D. (2019). Effects of Financial Education and Financial Literacy on Creative Entrepreneurship: A Worldwide Research. Educ. Sci..

[95] Welch V., Petticrew M., Petkovic J., Moher D., Waters E., White H., Tugwell P. (2016). The PRISMA-Equity Bellagio group. Extending the PRISMA statement to equity-focused systematic reviews (PRISMA-E 2012): Explanation and elaboration. J. Dev. Eff..

[96] Abad-Segura E., Cortés-García F.J., Belmonte-Ureña L.J. (2019). The sustainable approach to corporate social responsibility: A global analysis and future trends. Sustainability.

[97] Duque-Acevedo M., Belmonte-Ureña L.J., Cortés-García F.J., Camacho-Ferre F. (2020). Agricultural waste: Review of the evolution, approaches and perspectives on alternative uses. Glob. Ecol. Conserv..

[98] González-Zamar M.-D., Ortiz Jiménez L., Sánchez Ayala A., Abad-Segura E. (2020). The Impact of the University Classroom on Managing the Socio-Educational Well-being: A Global Study. Int. J. Environ. Res. Public Health.

[99] Prathap G. (2013). Quantity, quality, and consistency as bibliometric indicators. J. Assoc. Inf. Sci. Technol..

[100] Assefa S.G., Rorissa A. (2013). A bibliometric mapping of the structure of STEM education using co-word analysis. J. Am. Soc. Inf. Sci. Technol..

[101] Gingras Y. (2010). Mapping the structure of the intellectual field using citation and co-citation analysis of correspondences. Hist. Eur. Ideas.

[102] Van Eck N.J., Waltman L. (2009). Software survey: VOSviewer, a computer program for bibliometric mapping. Scientometrics.

[103] Bi X. (2017). Quality open access publishing and registration to Directory of Open Access Journals. Sci. Ed..

[104] Stiefel B.L. (2018). Rethinking and revaluating UNESCO World Heritage Sites. J. Cult. Herit. Manag. Sustain. Dev..

[105] Bergman Z., Bergman M., Fernandes K., Grossrieder D., Schneider L. (2018). The Contribution of UNESCO Chairs toward Achieving the UN Sustainable Development Goals. Sustainability.

[106] UNIBILITIY, University Meets Social Responsibility. http://www.eucen.eu/post/unibility.

[107] Hartley J. (2011). Refereeing academic articles in the information age. Br. J. Educ. Technol..

[108] Abramo G., D’Angelo C.A., Di Costa F. (2019). Diversification versus specialization in scientific research: Which strategy pays off?. Technovation.

[109] Dragicevic A.Z. (2018). Deconstructing sustainability. Sustain. Dev..

[110] Parkin S., Sommer F., Uren S. (2003). Sustainable development: Understanding the concept and practical challenge. Eng. Sustain..

[111] Gramatakos A.L., Lavau S. (2019). Informal learning for sustainability in higher education institutions. Int. J. Sustain. High. Educ..

[112] Nørreklit L., Jack L., Nørreklit H. (2019). Moving towards digital governance of university scholars: Instigating a post-truth university culture. J. Manag. Gov..

[113] Wu Y., Zhang K., Xie J. (2020). Bad Greenwashing, Good Greenwashing: Corporate Social Responsibility and Information Transparency. Manag. Sci..

[114] Lukinović M., Jovanović L. (2019). Greenwashing—Fake green/environmental marketing. Fundam. Appl. Res. Pract. Lead. Sci. Sch..

[115] Mowat J., Jopling M. (2019). Management in Education: Reflective piece draft guidelines. Manag. Educ..

[116] McGowan V. (2019). Higher Education Institutional Transparency of General Education Competencies, Assessment Measures, and Analysis of Assessment Data. J. Gen. Educ..

[117] Westerman J.W., Rao M.B., Vanka S., Gupta M. (2020). Sustainable human resource management and the triple bottom line: Multi-stakeholder strategies, concepts, and engagement. Hum. Resour. Manag. Rev..

[118] Yoda N., Kuwashima K. (2019). Triple Helix of University–Industry–Government Relations in Japan: Transitions of Collaborations and Interactions. J. Knowl. Econ..

[119] EuroHealthNet. https://eurohealthnet.eu/.

[120] CHAIN. Centre for Global Health Inequalities Research. https://www.ntnu.edu/chain#/view/about.

[121] Gerner M. (2019). Assessing and managing sustainability in international perspective: Corporate sustainability across cultures—Towards a strategic framework implementation approach. Int. J. Corp. Soc. Responsib..

[122] Harini C., Priyanto S.H., Ihalauw J.J., Andadari R.K. (2020). The role of ecological innovation and ecological marketing towards green marketing performance improvement. Manag. Entrep. Trends Dev..

[123] Bastas A., Liyanage K. (2019). Setting a framework for organisational sustainable development. Sustain. Prod. Consum..

[124] Gupta S., Agrawal R. (2017). Environmentally Responsible Consumption: Construct Definition, Scale Development, and Validation. Corp. Soc. Responsib. Environ. Manag..

